# Supplementary material for: A comprehensive manually-curated compendium of bovine transcription factors
Source: Sci Rep. 2018 Sep 13;8:13747. doi: 10.1038/s41598-018-32146-2 (PMC6137171; doi:10.1038/s41598-018-32146-2)
Supplement: Supplementary file 1 — Supplementary Figures [file 41598_2018_32146_MOESM1_ESM.pdf]

A comprehensive manually-curated compendium of bovine transcription factors

Marcela M de Souza[1,2], Adhemar Zerlotini[3], Ludwig Geistlinger[2], Polyana C Tizioto[4], Jeremy F Taylor[5], Marina IP Rocha[1], Wellison JS Diniz[1], Luiz L Coutinho[6], Luciana CA Regitano[2*]

[1]Post-graduation Program of Evolutionary Genetics and Molecular Biology, Federal University of São Carlos, São Carlos, São Paulo, 13560-970, Brazil
[2]Animal Biotechnology, Embrapa Pecuária Sudeste, São Carlos, São Paulo, 13560-970, Brazil
[3]Bioinformatic Multi-user Laboratory, Embrapa Informática Agropecuária, Campinas, São Paulo, 70770-901, Brazil
[4]NGS Genomic Solutions, Piracicaba, São Paulo, Brazil
[5]Division of Animal Science, University of Missouri, Columbia, Missouri, 65211-5300, USA
[6] Functional Genomic Center, University of São Paulo, Piracicaba, São Paulo, 13418-900 Brazil

* Correspondence: Tel: +55(16)34115637; Email: luciana.regitano@embrapa.br

Content:

- Supplementary Figures
- Supplementary Table legends
- References

SUPPLEMENTARY FIGURES

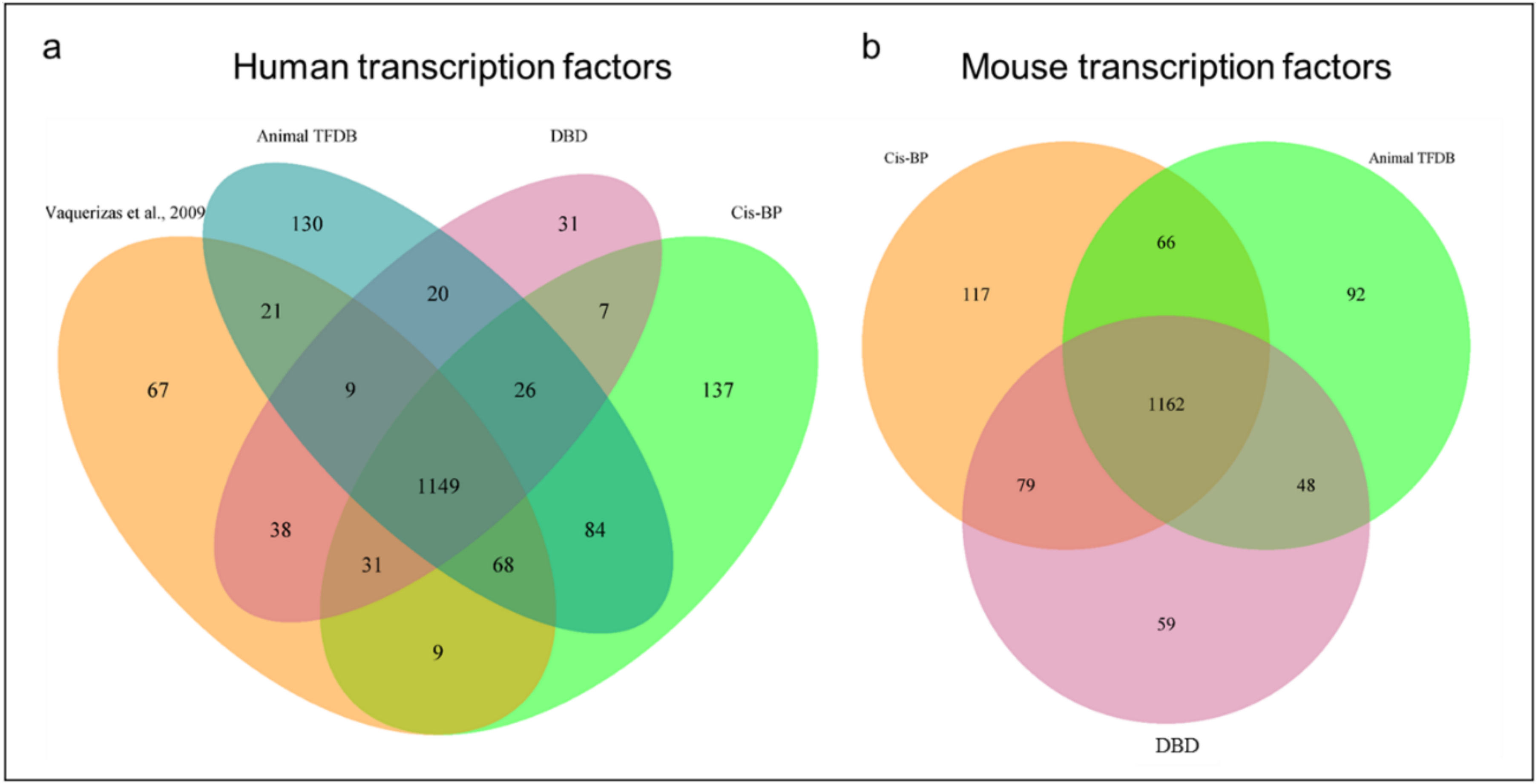

Supplementary Figure S1. Human and Mouse existing transcription factors (TFs) databases. Venn diagram comparing TFs from existing transcription factors (TFs) databases. (a) Human TFs from Vaquerizas *et al*. [1], Animal TFDB [2], DBD [3] and Cis-BP [4]. (b) Mouse TFs from Cis-BP, DBD, and TFDB.

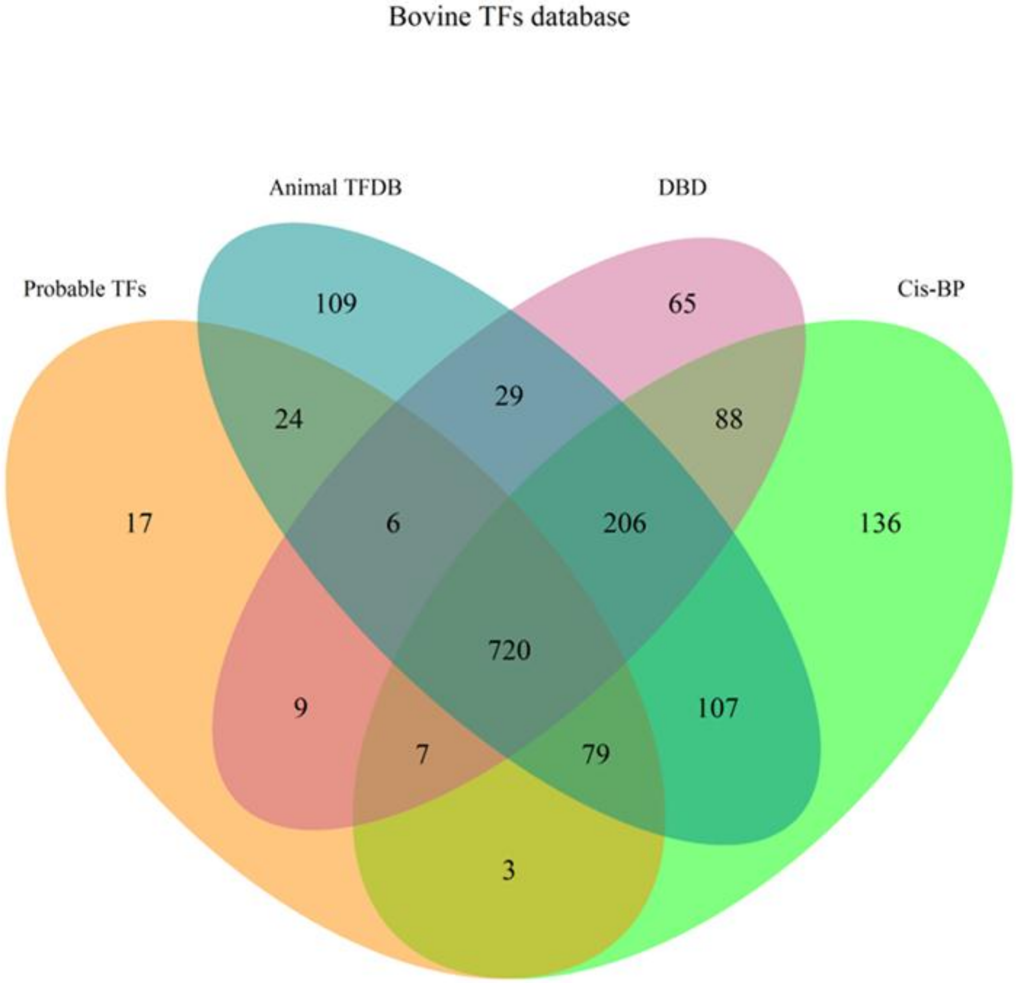

Supplementary Figure S2. Bovine existing transcription factors (TFs) databases. Venn diagram comparing bovine TFs in our compendium with TFs listed for bovine in three existing TF databases: Animal TFDB [2], DBD [3] and Cis-BP [4].

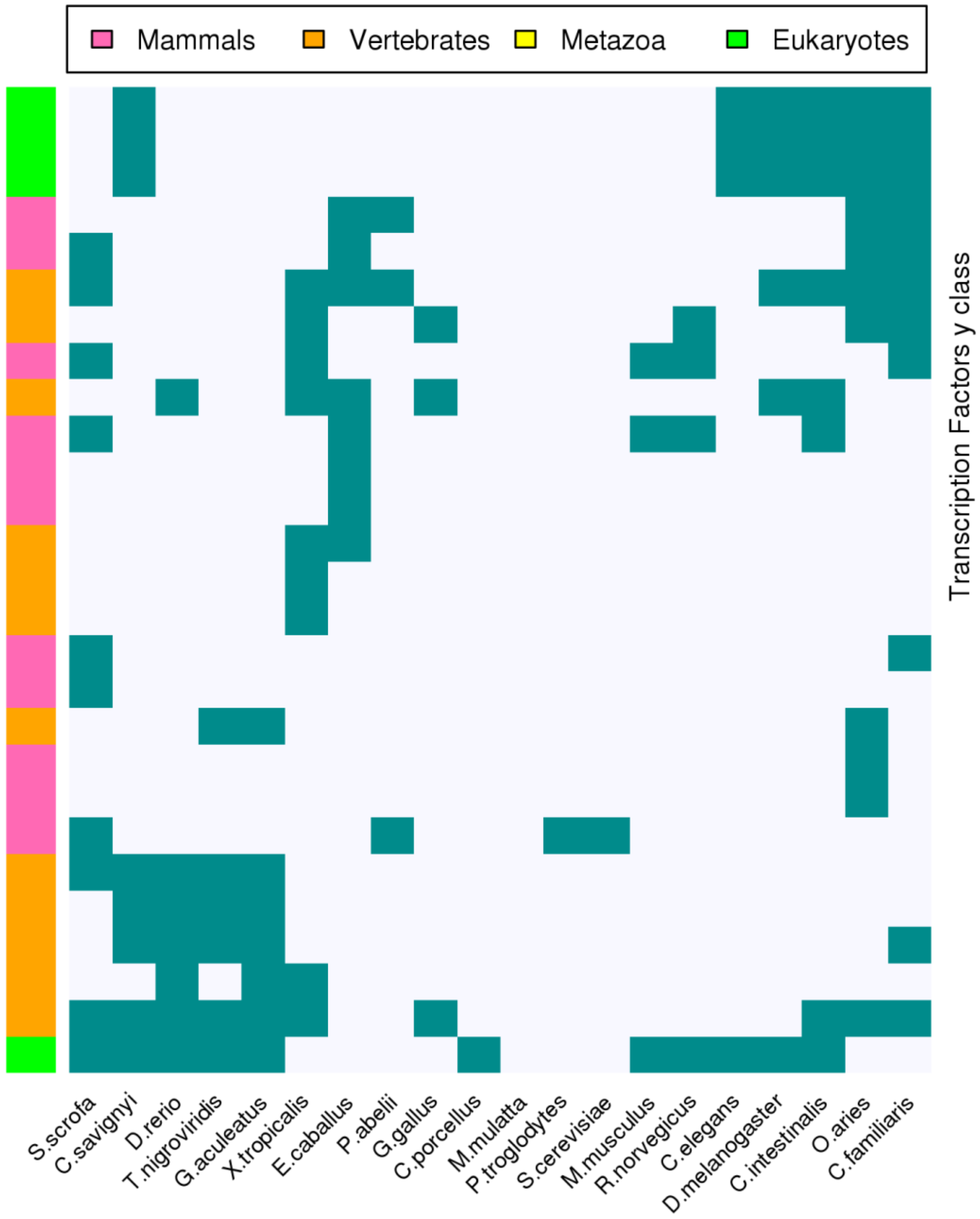

Supplementary Figure S3. Heat map showing the conservation of bovine TFs without human orthologues across 20 eukaryotic species. Rows represent the TFs and columns the species; both are hierarchically clustered according to the presence (orange) or absence (white) of orthologues in the respective species. The color bar on the right indicates whether TFs are predominantly present in mammal (pink), vertebrate (orange), Metazoa (yellow) or all analyzed eukaryotes (green).

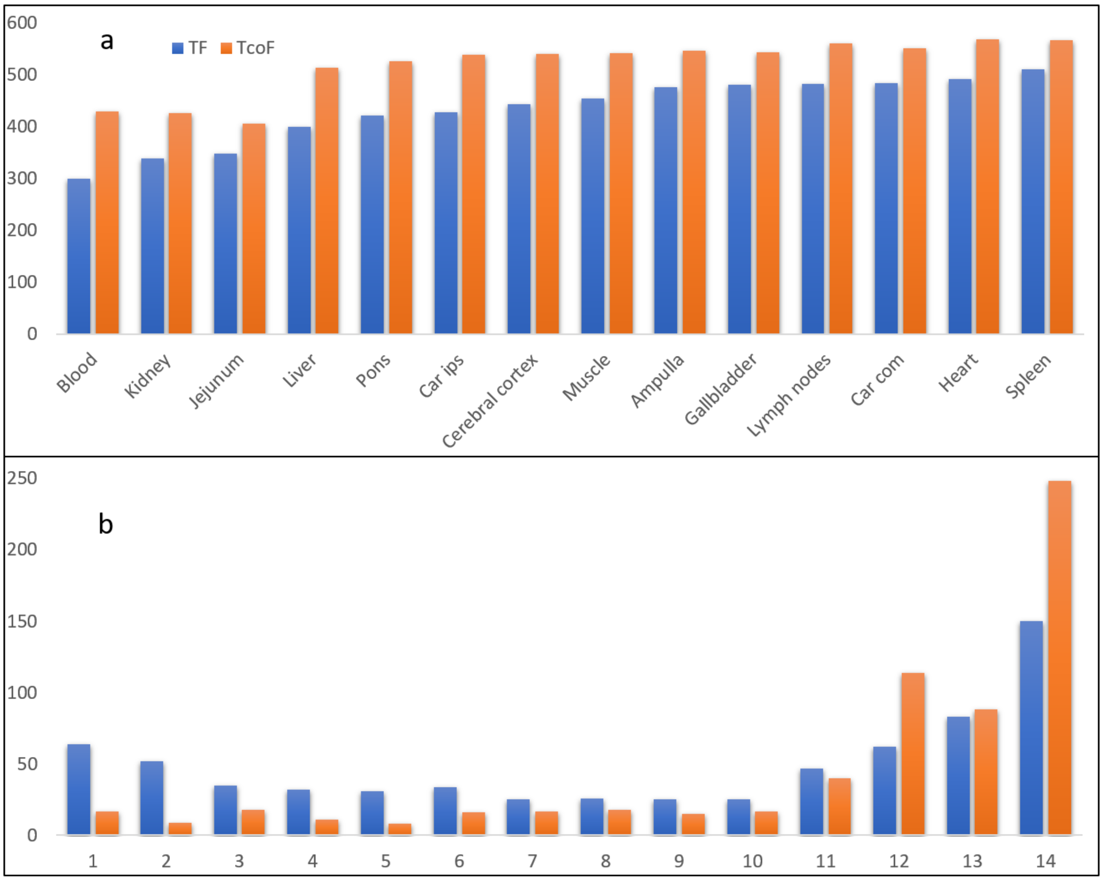

Supplementary Figure S4. (a) Number of TFs and TcoFs, independently determined to be expressed across all tissues. (b) Number of tissues in which TFs and TcoFs are independently determined to be expressed.

SUPPLEMENTARY TABLE LEGENDS

Supplementary Table S1. Updates on evidence for transcriptional activity. TFs previously classified as “b” or “c” that were reclassified as “a” duo to new evidence of transcriptional activity in the literature. The list contains accompanying information including: Ensembl gene IDs, human orthologue gene, orthology type, bovine TFs repertoire classification, Vaquerizas *et al*. [1] TF classification, literature references of experimentally evidences.

Supplementary Table S2. List of Interpro DNA-binding domains and families used to characterise the bovine TFs repertoire.

Supplementary Table S3. Bovine TFs with BLAST to “a” or “b” class human TF. The list contains accompanying information including: Ensembl gene IDs, HGNC identifiers, bovine TFs repertoire classification, Vaquerizas *et al*. [1] TF classification, BLAST results.

Supplementary Table S4. Final list of all genes analyzed and the bovine TFs classification. List of genes classified as “a”, “b”, “c”, “x”, ”y”. The list contains accompanying information including: Ensembl gene IDs, HGNC identifiers, human orthologue gene, orthology type and tissue expression if any.

Supplementary Table S5. *Bos taurus* TcoF repertoire. List of TcoF-encoding loci classified as “high-confident” or three “hypothetical” groups. The list contains accompanying information including: Ensembl gene IDs, HGNC identifiers, Uniprot IDs, human orthologue gene, orthology type, bovine transcription factor ID pair, reliability classification and TcoF tissue expression if any.

Supplementary Table S6. FPKM *vs* tissue for each TF expressed in at least one tissue.

Supplementary Table S7. FPKM *vs* tissue for each TcoF expressed in at least one tissue.

Supplementary Table S8. TF-TcoF Co-expression in 14 bovine tissues.

REFERENCES

1. Vaquerizas, J. M., Kummerfeld, S. K., Teichmann, S. A. & Luscombe, N. M. A census of human transcription factors: function, expression and evolution. *Nat. Rev. Genet.* **10,** 252–263 (2009).

2. Zhang, H. M. *et al.* AnimalTFDB 2.0: A resource for expression, prediction and functional study of animal transcription factors. *Nucleic Acids Res.* **43,** D76–D81 (2015).

3. Wilson, D., Charoensawan, V., Kummerfeld, S. K. & Teichmann, S. A. DBD - Taxonomically broad transcription factor predictions: New content and functionality. *Nucleic Acids Res.* **36,** (2008).

4. Weirauch, M. T. *et al.* Determination and Inference of Eukaryotic Transcription Factor Sequence Specificity. *Cell* **158,** 1431–1443 (2014).
